# When the Damage Is Done: Injury and Repair in Thymus Function

**DOI:** 10.3389/fimmu.2020.01745

**Published:** 2020-08-12

**Authors:** Sinéad Kinsella, Jarrod A. Dudakov

**Affiliations:** ^1^Program in Immunology, Clinical Research Division, Fred Hutchinson Cancer Research Center, Seattle, WA, United States; ^2^Immunotherapy Integrated Research Center, Fred Hutchinson Cancer Research Center, Seattle, WA, United States; ^3^Department of Immunology, University of Washington, Seattle, WA, United States

**Keywords:** endogenous thymic regeneration, immune restoration, T cell reconstitution, thymic epithelial cells, BMP4, IL-22

## Abstract

Even though the thymus is exquisitely sensitive to acute insults like infection, shock, or common cancer therapies such as cytoreductive chemo- or radiation-therapy, it also has a remarkable capacity for repair. This phenomenon of endogenous thymic regeneration has been known for longer even than its primary function to generate T cells, however, the underlying mechanisms controlling the process have been largely unstudied. Although there is likely continual thymic involution and regeneration in response to stress and infection in otherwise healthy people, acute and profound thymic damage such as that caused by common cancer cytoreductive therapies or the conditioning regimes as part of hematopoietic cell transplantation (HCT), leads to prolonged T cell deficiency; precipitating high morbidity and mortality from opportunistic infections and may even facilitate cancer relapse. Furthermore, this capacity for regeneration declines with age as a function of thymic involution; which even at steady state leads to reduced capacity to respond to new pathogens, vaccines, and immunotherapy. Consequently, there is a real clinical need for strategies that can boost thymic function and enhance T cell immunity. One approach to the development of such therapies is to exploit the processes of endogenous thymic regeneration into novel pharmacologic strategies to boost T cell reconstitution in clinical settings of immune depletion such as HCT. In this review, we will highlight recent work that has revealed the mechanisms by which the thymus is capable of repairing itself and how this knowledge is being used to develop novel therapies to boost immune function.

## Introduction

Generation of a diverse but tolerant T cell repertoire, which is critical for adaptive immune function, is dependent on the development and maturation of T cell precursors in the thymus. The process of T cell development is reliant on the interactions with the stromal microenvironment, comprised of highly specialized thymic epithelial cells (TECs), endothelial cells (ECs), mesenchymal cells, dendritic cells (DCs) and macrophages. However, despite its importance for generating and maintaining T cells, thymic function is extremely sensitive to acute damage such as that caused by everyday insults like stress and infection, as well as more profound injuries such as that caused by cytoreductive therapies. Nevertheless, the thymus also has a remarkable capacity to regenerate itself from these acute injuries (1, 2), although until recently this phenomena has been largely unstudied. However, despite its crucial function, the ability of the thymus to facilitate efficient T cell generation deteriorates progressively with age (3, 4); which considerably hampers the ability of the thymus to respond to acute insults. Age-related thymic atrophy and immunosenescence are hallmarks of immune aging and ultimately lead to a constriction of the TCR repertoire (5), decreased naïve T cells and accumulation of memory T cells in the periphery; and chronic low-grade inflammation termed “inflamm-aging,” all conferring insufficient protective responses to pathogens and neoantigens (6–8). Together, these acute and chronic thymic injuries underlie prolonged immune deficiency associated with multiple conditions including the conditioning required for hematopoietic cell transplantation (HCT) and cytoreductive cancer treatments such as radio- and chemo-therapies.

**Figure 1:**
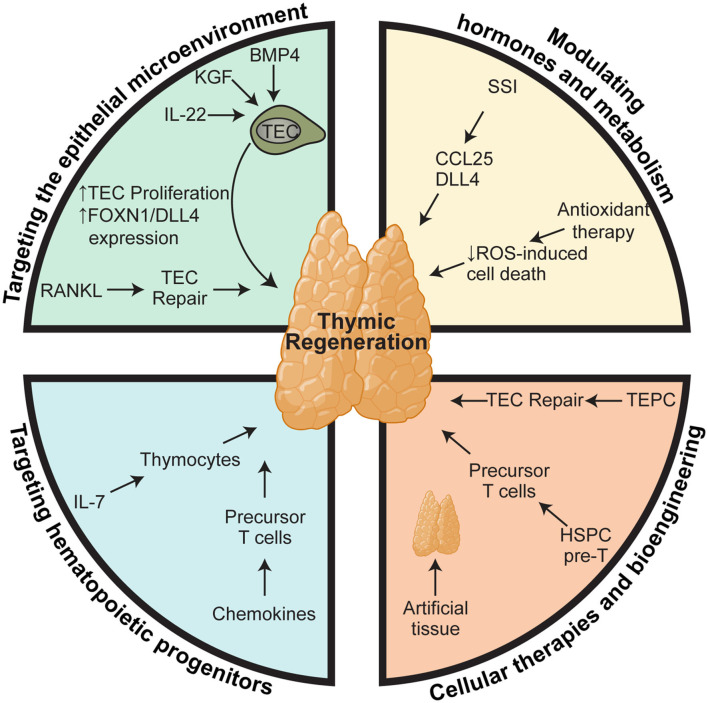
Therapeutic approaches for boosting thymus function. Regenerative therapies to boost thymic function after acute damage or to reverse age-related involution can be broadly stratified into four subgroups based on their cellular or molecular targets: (1) targeting the epithelial microenvironment that supports thymopoiesis; (2) targeting the precursors that provide the supply of developing thymocytes; (3) modulation of hormones and metabolism; and (4) cellular therapies and bioengineering. However, within each of these therapeutic modalities there are key nexus points at which they act mechanistically. One approach relies on stimulating TEC function, such as IL-22, BMP4, KGF, RANKL, SSI, which act by either promoting survival, proliferation, differentiation, or expression of key thymopoietic molecules like DLL4 and KITL. In contrast, approaches such as administration of exogenous IL-7 and chemokine therapies target T cell precursors to promote their migration, proliferation, and differentiation directly. Similarly, many of the bioengineering approaches have sought to recapitulate these same functions such as providing TEC signals or a ready supply of T cell precursors. Elements of the figure were generated using Biorender.com.

Given the poor outcomes that are associated with deficient T cell immunity, there is a clear clinical need for therapies that can boost thymic function in periods of acute injury or reverse age-related thymic involution. In this review, we will outline what we know about how the thymus is damaged during different modalities of insult and the work that has been done to develop therapeutic strategies to boost thymic function; either ensuing acute insult such as following HCT, or in aged individuals to boost responses to vaccines or immunotherapy (Figure 1).

## Acute Damage and Endogenous Regeneration in the Thymus

### Everyday Insults: Stress and Infection

Thymic involution is a routine response to acute insult incurred by multiple triggers including emotional and physical distress, malnutrition, and opportunistic bacterial and viral infections. These can be modeled using approaches such as synthetic corticosteroid treatment, such as dexamethasone (9); nutrient depletion (10); sex steroid treatment (11); and several viral and bacterial infection models. While acute thymic involution results primarily from the loss of cortical thymocytes (12, 13); in cases of chronic atrophy, such as that induced by age-related thymic decline, thymocyte loss is preceded by the loss of Foxn1+ TECs, resulting in the functional decline the TEC compartment and the initiation of age-related thymic atrophy (14, 15).

Continuous export of naïve cells from the thymus, or recent thymic emigrants (RTEs), is essential for effective immune response to acute and chronic infections (16, 17). However, most acute bacterial or viral infections result in acute thymic atrophy, largely due to intense lymphocyte depletion as a result of increased apoptosis of thymocytes and interference with thymocyte development (18–21); which can, at least partially, be attributed to the increased induction of IFNγ from activated CD8+ T cells (22) and Natural Killer (NK)-driven responses (23). Most of the studies looking at viral infection-related thymic function have concentrated on HIV, which leads to several modes of thymic dysfunction including thymic atrophy, reduced thymic output, reduced export of immature thymocytes and disruption of the thymic microenvironment (24–26). Notably, effective response to anti-retroviral therapies was found to depend on competent thymic function, with enhanced function in HIV-infected children with higher basal levels of thymic function (27), in contrast with infected adults who have a reduced thymic output and output decreased CD4+ T cells (28, 29). Moreover, in addition to viral load, quantification of CD4+ RTE has long been known as a suitable marker for HIV disease progression, and a recent study has demonstrated the use of RTE CD4+ T cells as a marker of perinatal HIV infection in infants (30); further strengthening the link between viral infection, efficient thymic function, and therapeutic implications of thymic atrophy.

Although less well studied, bacterial infections also have negative effects on thymic function, primarily by enhancing thymocyte apoptosis. *Streptococcus suis* infection promotes thymic atrophy specifically by inducing increased activation of pro-apoptotic pathways and apoptotic cell death in thymocytes (31); while *Mycobacterium tuberculosis* infection also induces thymic atrophy (32), possibly by regulating glucocorticoid levels and in this way impact on homeostatic endocrine-immune communication (33). In fact, glucocorticoids are central to many acute forms of thymic involution (34, 35), directly inducing the apoptosis of CD4+CD8+ DP thymocytes, which preferentially express the glucocorticoid receptor (36).

Metabolic distress due to lack of nutrients, primarily glucose, leads to an attenuation of thymic function, with perturbed thymopoiesis in non-obese diabetic (NOD) mice (37), and reduced thymic atrophy with glucose supplementation in models of mitochondrial dysfunction (38).

### Cytoreductive Therapies

Most therapies used in cancer treatments are cytoreductive, such as chemotherapy or radiation. One prominent example of this is that the pre-conditioning regimens required for successful HCT result in profound injury to the thymus, and, in contrast to the relatively early recovery of platelets, erythrocytes, and leukocytes involved in innate immunity, recipients of an HCT experience prolonged post-transplant deficiency in the recovery of adaptive immunity, especially T cell immunity. This delayed T cell reconstitution can last a year or more due to a delay in full recovery of function and T cell repertoire (39–41). Moreover, post-transplant T cell deficiency is associated with an increased risk of infections (39, 40, 42, 43), relapse of malignancy (44), and the development of secondary malignancies (45–50). In fact, infection and relapse account for >50% of mortality following allogeneic-HCT (allo-HCT) (51). T cell reconstitution after transplant is critically dependent on the thymus (39, 41, 47, 52–58) and thymic function pre-transplant can have a significant impact on clinical outcomes. Similarly, damage caused by cytoreductive chemotherapy results in significant thymic damage and can lead to a profoundly delayed recovery of T cells (45, 59). In mouse models of chemotherapy, in addition to almost complete depletion of thymocytes, there was also a severe depletion of TECs, most prominently MHCII^hi^ TECs (60); likely as they are the most highly proliferating TEC subset (61, 62). Specifically, genotoxic stress caused by chemotherapy leads to senescence in the thymic stromal compartment and the induction of an inflammatory environment in the thymus with endothelial cell secretion of IL-6, generating a chemoresistant niche that is cytoprotectant to certain cancer cells, such as lymphoma and melanoma (63, 64). Accompanying the damage caused by cytoreductive conditioning, the risk of further thymic damage caused by Graft-vs.-Host Disease (GVHD) is significant in the context of an allo-HCT. In fact, the thymus is a particularly sensitive GVHD target organ and presents pathological features even in the context of subclinical GVHD (65–67). Furthermore, there is likely a link between acute GVHD-mediated thymic damage and the formation of chronic GVHD, which may in part be a failure for tolerance induction (68–70).

## Strategies of Boosting Thymic Function I: Targeting Non-Hematopoeitic Cells

Given the sensitivity of thymic function to negative stimuli, even everyday insults, a reparative capacity is crucially important for renewal of immune competence. In fact, this capacity of the thymus to regenerate itself has been known for longer than even the immunological function of the tissue was discovered (71, 72); however, until recently the mechanisms underlying this process have been poorly understood. One approach to developing therapies to enhance thymic function has come from exploiting these pathways of endogenous regeneration. Many of these pathways that mediate endogenous regeneration have been found to be effective for exogenous regeneration in periods of acute and profound injury such as that caused by cytoreductive chemotherapy and γ-radiation. Interestingly, many of these pathways specifically target TECs to mediate regeneration.

### Interleukin-22

Although the phenomenon of endogenous thymic regeneration has been known for over almost a 100 years, it was not until recently that pathways mediating this regeneration have been described. The first of these was centered around the production of Interleukin-22 (IL-22), a member of the IL-10 family that typically targets non-hematopoietic cells such as epithelial cells and fibroblasts (73). In this regenerative network, acute damage to the thymus (and specifically the depletion of thymocytes) triggers the release of Interleukin-23 (IL-23) from dendritic cells, which induces the production of IL-22 by a group 3 innate lymphoid cells (2, 74–76). Expression of IL-22R in the thymus is lacking on thymocytes but detected in both cTECs and mTECs populations (2). IL-22 acts on TECs to mediate repair but the specific molecular mechanisms are not clear. In addition to the thymus, IL-22 also has a major role in the regeneration of epithelial cells in a diverse range of tissues including gut, lung, skin, breast, and kidney (77). The IL-22 receptor is a type 2 cytokine receptor, and a heterodimer formed of two subunits: IL-10 receptor 1 (IL-10R1) and IL-22 receptor A2 (IL-22RA2) (78). IL-22 receptor binding induces intracellular inactivation of the Jak1/Tyk2 complex which further allows downstream signaling and phosphorylation of Signal Transducer and Activator of Transcription (STATs) 1, 3, and 5, with a preference for STAT3 phosphorylation (79), including in TECs (2) which is consistent with the upregulation of Foxn1 concurrently with IL-22 in the thymus (76), and the importance of STAT3 for TEC maintenance (80). Furthermore, Ruxolitinib, a chemotherapeutic agent that inhibits Jak1 signaling also prevents thymic regeneration after injury (81).

Similar to other tissues (77), IL-22 is not required for the formation or maintenance of the thymus under steady-state physiological conditions; however, it has a key role in driving thymic regeneration after injury, by acting directly on TECs to induce survival and proliferation, potentially via regulation of Foxn1 expression (2, 76). Of note, both the numbers of innate lymphoid cells (ILC) 3 and IL-22 levels were decreased in the thymus and gut of mice with GVHD (74, 82), suggesting that ILCs are a target of alloreactive cells and this depletion likely causes a failure to repair after damage. Due to the diverse pathophysiological roles of IL-22, and the key role in epithelial cell regeneration, modulation of the IL-22-IL22R system is an attractive therapeutic target. In fact, a clinical trial is currently underway to assess the efficacy and safety of administration of IL-22 in combination of systemic corticosteroids to limit the effects of GVHD after hematopoietic stem cell transplantation, with secondary readouts to assess T cell reconstitution (NCT02406651).

### Bone Morphogenic Protein 4

Although the role of thymic BMP4 and the endogenous BMP4R antagonist Noggin have been well-described in thymic development (83, 84), only recently has BMP4 been described as a regulator of thymic regeneration after acute injury (85). In the thymus, the source of BMP4 is fibroblasts and endothelial cells (ECs) (85). ECs are a highly radio-resistant population of cells in the thymus (85, 86) and are unique in their ability to induce BMP4 production in response to injury. Importantly, thymic expression of both *Bmpr1a* and *Bmpr2* were identified on TEC populations, with a higher expression of the non-redundant *Bmpr2* on cTECs (85); consistent with BMP4-induced expression of FOXN1 and its downstream target delta-like 4 (DLL4) specific to cTECs (85, 87). Although the importance for FOXN1 and DLL4 for the development of TECs and thymocytes, respectively, has been well studied (88, 89), recent findings have also highlighted their importance for thymic regeneration, with intrathymic concentration of DLL4 profoundly impacting on thymic size (90), and reports suggesting that the induction of FOXN1 can counteract age-associated thymic involution (91), acute damage (85), and thymic damage post-transplantation (92). While much of the role of BMP4 seems to be mediated by induction of the FOXN1/DLL4 axis, given the requirement for BMP4 in *in vitro* differentiation of TECs from multipotent progenitors (93–95), it is possible that an alternate mechanism may be by stimulating bipotent progenitors present in the adult thymus (96–99). Unfortunately, the preclinical studies assessing BMP4 have yet to successfully treat mice with recombinant protein, a therapeutic strategy has been developed that utilizes a technique of allowing for the propagation and expansion of tissue-specific ECs that can be transplanted and mediate regeneration across multiple tissues (85, 100–105). In the thymus, it was found that this therapeutic cellular strategy was dependent on the expression of BMP4 by transplanted ECs (85).

### Keratinocyte Growth Factor

Keratinocyte growth factor (KGF, also known as FGF-7), is a fibroblast growth factor and acts as a mitogen targeting TECs, inducing epithelial proliferation in several organs (106–108). In the thymus, KGF is primarily produced by mature αβ^+^ thymocytes and feeds back to facilitate the proliferation and expansion of mTECs via the activation of p53 and NF-κB pathways (106, 108), preserving the thymic cytoarchitecture. Of note, KGF is also produced by thymic fibroblasts (108). Expression of the KGF receptor, fibroblast growth factor receptor-2 of the IIIb variant (FgfR2IIIb), is limited to TECs (109), and FgfR2-IIIb^−/−^ mice have defective thymopoiesis and reduced cellularity, accounted for specifically by a reduction in TECs (110). KGF modulates TEC functionality by negatively regulating the levels several gatekeepers of positive selection, such as MHC-II invariant chain (Ii), and cathepsin L (CatL) (108), and acts on TECs to produce several cytokines that act directly on thymocytes to facilitate maturation, such as bone morphogenic protein 2 (BMP2), BMP4, Wnt5b, and Wnt10b (109).

Under normal physiological conditions, KGF can enhance thymic cellularity by increasing the number of early thymic progenitors (ETPs) equating to an enhanced number of engraftment niches, and increased TEC proliferation (109). Although it was shown that KGF is not essential in uninjured conditions (110, 111), studies using *KGF*^−/−^ mice demonstrated the critical role of KGF on thymus function and immune reconstitution after insult, modeled by both syngeneic and allogeneic bone marrow transplant (112). The same study showed that exogenous administration of recombinant KGF enhanced thymopoiesis in young and middle-aged mice, and attenuated the negative effects of acute thymic injury, such as that caused by dexamethasone treatments, cyclophosphamide, and irradiation, highlighting an extremely attractive therapeutic approach to efficiently facilitating immunocompetence after damage. Additionally, exogenous KGF administration improved post-transplantation T cell reconstitution. Furthermore, pre-conditioning with KGF prior to bone marrow transplantation reduces GVHD in mouse models by protecting against epithelial injury (113). However, a recent clinical trial noted a reduction in thymopoiesis in lymphopenic patients following administration of KGF (114), highlighting that more studies need to be carried out before KGF can be used across the board as a therapeutic regulator of thymic regeneration.

### RANKL

Receptor activator of nuclear factor kappa-B ligand (RANKL), a member of the Tumor necrosis factor (TNF) superfamily, is implicated in multiple physiological roles in the periphery, primarily in bone biology (115). RANKL has an essential role in the thymus as a potent inducer of epithelial cell differentiation by regulating the key mTEC transcription factor Aire (116). In this way, RANKL governs the maturation of Aire^−^ mTECs to Aire^+^ mTECs which subsequently present MHC-II peptides that drive the elimination of self-reactive T cells during negative selection (117). RANKL is non-redundant for fetal Aire+ mTEC development, and is produced during development by ILCs, and subsequently by subsets of thymocytes (116, 118–120); although absence of RANKL postnatally can be compensated for by other factors (121). Importantly, RANKL is increased in CD4+ thymocytes and ILCs after injury from the cytoreductive conditioning required prior to HCT, suggesting that RANKL plays a role in endogenous regeneration of the thymus (2, 122).

The prominent role of RANKL in mTEC biology points to the ability of RANKL to modulate thymic regeneration and output. RANKL administration shows an enhancement of thymic function after bone marrow transplantation by boosting TEC subsets, including TEC progenitor niches (122). Moreover, systemic administration of recombinant soluble RANKL (sRANKL) improved thymic medullary architecture in RANKL deficient mice (123), and transgenic mice overexpressing human sRANKL, or mice lacking the soluble RANKL receptor OPG, have an enlarged thymic medulla with increased numbers of Aire+ mTECs (119, 124, 125), highlighting a therapeutic platform for the use of recombinant RANKL as a therapeutic for thymus regeneration.

## Strategies of Boosting Thymic Function II: Targeting Hematopoeitic Cells

Given the fact that Cell development requires the input of hematopoietic progenitors, and the fact that the supply of those progenitors is severely limited after acute injury (126, 127), one approach to promoting thymic function is to directly stimulate precursor populations; either in the BM or thymus.

### Bone Marrow Progenitors

Several approaches have been attempted that seeks to improve thymic function by stimulating the function of bone marrow hematopoietic progenitors. For instance, preclinical studies have shown that administration of Flt3L can also enhance both thymic dependent and independent T cell reconstitution (128, 129). The effects of Flt3L are predominantly due to an expansion in Flt3^+^ progenitors in the BM (130). However, increases in T cell reconstitution can be at the expense of B-lymphopoiesis which is significantly declined with exogenous Flt3L administration and, in particular, its effects on the EPLM subset of BM progenitors (131, 132).

Chemokines are key regulators of thymopoiesis, facilitating thymic population and intrathymic cell migration. Importantly, as the thymus does not contain long term progenitors that would enable self-renewal, repopulation of the thymus requires continuous recruitment of T cell progenitors (133). CCL25 (with its receptor CCR9) and CCL21 (with its receptor CCR7) play an important role in thymic colonization with hematopoietic progenitors (134). Interestingly, chemokine therapy, whereby bone marrow progenitors received CCL25 and CCL21 treatment prior to transplant, rescues thymic homing of progenitors which is otherwise suppressed in irradiated mice (86).

### Thymic T Cell Precursors

While there are several approaches that have been postulated that target thymic precursor cells the most prominent and developed of these is with the lymphopoietic cytokine interleukin-7 (IL-7). IL-7 has a non-redundant role as a survival molecule in lymphoid tissues in mice and humans, most importantly in the thymus where IL-7 is critical for appropriate thymocyte development. An elegant study by Shitara et al. (135) showed that specific deletion of IL-7 in TECs resulted in the profound reduction in αβ and γδ T cells; and mice deficient for *Il-7* have a peripheral loss of γδ T cells, a significant reduction in αβ T cells (136), an absence of innate lymphoid cell subsets (137), and disorganization of lymphoid tissue (138). Moreover, mice lacking *Il-7* have a reduced number of DN2 or DN3 cells (139, 140), essentially creating a thymic block and limiting the progression thymocytes to maturity.

IL-7 is produced primarily by non-hematopoietic stromal cells such as TECs and signals by binding to the heterodimeric IL-7 receptor (IL-7R), comprised of IL-7R? (also known as CD127) and the cytokine receptor γ-chain (also known as CD132), and induces an anti-apoptotic pro-survival signaling cascade via the activation of phosphoinositide 3-kinase (PI3K) and the Janus Kinase (JAK)-STAT pathway. The expression of IL-7R? on developing thymocytes occurs in a cyclical pattern, with expression seemingly dependent on the fluctuating need for IL-7 signaling at different stages of thymocyte maturation (141), demonstrated by absence in the earliest T cell progenitors, expression at later DN stages, absence at the DP stage and re-expression in SP thymocytes.

These critical roles of IL-7 in both thymocyte development and in peripheral T cell homeostasis (142) reveal IL-7 as a strong therapeutic candidate to enhance T cell development and activation. Clear evidence exists for the therapeutic potential of IL-7 administration on thymic regeneration, centered on the beneficial effects of IL-7 on increasing progenitor T cells in the thymus and subsequently expanding circulating naïve T cells in viral infection setting (143); however, IL-7 therapy only transiently increased naïve T cells in the aged setting in rhesus macaques, with a more prominent and long lasting effect in the memory T cell compartment (144). Although recombinant IL-7 immunotherapy has had some success in clinical trials for treating septic shock (145), infection (146), and cancer remission (147), along with some early promise in the setting of HCT (148), further studies are necessary to identify a strategy for thymus-dependent IL-7 therapy.

## Strategies of Boosting Thymic Function III: Modulation of Hormones and Metabolism

Given the impact of sex steroids on thymic function (149, 150), surgical or chemical ablation of sex steroids has been a well-studied means of boosting thymic function (58, 151). In fact, sex steroid inhibition (SSI) has been shown to promote thymic function in young as well as old mice, and enhances reconstitution after acute insult such as chemotherapy or HCT (60, 152–154). Furthermore, given that SSI is a standard and approved therapy for prostate cancer, thymic function has been assessed in prostate cancer and after HCT and significant improvement observed (155, 156). Although whole organismal ablation of sex steroids will understandably have systemic effects, and the specific means by which SSI improves thymic function are not yet clear, several putative mechanisms have been proposed. In particular, SSI has been shown to (1) promote lymphoid potential and overall function of hematopoietic stem and progenitor cells (2, 152, 157, 158) induce the expression of CCL25 (159), which promotes the importation of hematopoietic progenitors from the circulation (3, 134, 160) induces the expression of the Notch ligand DLL4 (90). Interestingly, KGF was not required for the beneficial effects of SSI on thymus (154), and in fact combination therapies have shown great promise, with the combined KGF administration and androgen blockage with Lupron, revealing reduced epithelial damage and enhanced T cell reconstitution after bone marrow transplant in mice (161). However, it has also been reported that regrowth of the thymus can result in an increase in autoreactive T cells in the periphery, particularly in models of castration, reflecting a lack of synergy between quality and quantity of thymopoiesis (162).

In addition to sex steroids, several other hormones and metabolic components have been implicated in thymic function and their modulation has been shown to improve thymopoiesis, particularly in the aged. Administration of the appetite stimulating hormone Ghrelin led to improved thymic cellularity and thymic output in aged mice (163), and similarly oral zinc supplementation increased thymic cellularity in aged mice (164). Targeting accumulating reactive oxygen species with antioxidants has proven to be beneficial in protecting against age-related thymic atrophy, whereby treatment with the mitochondrial antioxidant SkQ1 reduced age-associated thymic atrophy and increased the number of CD4+ and CD8+ thymocytes (165). Similarly, Leptin, a peptide hormone secreted from adipose tissue, has a protective effect on thymopoiesis in LPS-treated mice and mice that had been starved, primarily due to rescue from metabolic defects including increased corticosterone levels (10, 166).

## Strategies of Thymic Regeneration IV: Cell Therapies and Bioengineering Approaches

### Hematopoietic Precursors

In addition to the use of growth factors and hormone modulation, several groups have been working on cellular therapies that may enhance thymic function. Given that some of the delay in T cell reconstitution is due to the limited supply of BM-derived progenitors (126), in addition to the time taken for development into a naïve lymphocyte from a transplanted HSC, early studies that concentrated on providing hematopoietic cells found that lymphoid precursors isolated from donor bone marrow could be used to boost thymic function when infused into a recipient at the time of HCT, giving an early boost to T cell development (167). To overcome the limited number of hematopoietic progenitors in BM, an alternate approach of using precursor T cell populations that have been expanded using *ex vivo* culture systems that use Notch-1 stimulation of hematopoietic precursor cells has been demonstrated (168–173). Using this regimen, adoptive transfer of T cell precursors into lethally irradiated allogeneic HCT recipients caused a significant increase in thymic cellularity and chimerism, as well as enhanced peripheral T and NK cell reconstitution compared with recipients of allogeneic hematopoietic stem cells only (168, 174–178).

### Thymic Epithelial Cells

In addition to the use of hematopoietic cells that can act as a boost of T cell precursors, another approach is to identify and isolate populations of thymic epithelial progenitor cells (TEPC). TEPC have been successfully isolated from fetal thymi and induced to generate a new thymus in athymic recipients (179–182), and neonatal TECs, or TECs derived from pluripotent progenitors can promote enhanced thymic function (183, 184). However, while there is evidence of a bipotent TEPC in the postnatal thymus (97–99), their capacity to self-organize as a whole organ like fetal TEPCs is limited. A TEC-like progenitor cell appropriate for this purpose has also been generated by direct conversion of embryonic fibroblasts by induced expression of the TEC transcription factor FOXN1 (185); although the efficacy of this therapy in a regeneration setting has not been investigated.

### Artificial Thymic Niches

Finally, there are also several approaches that do not rely on the endogenous thymus at all, but rather concentrate on *de novo* formation of whole organs *ex vivo* that can be transplanted into patients as required (186, 187). Although *in vivo* evidence of their efficacy is still only limited, several approaches have been used to generate artificial thymuses *ex vivo*, including decellularizing the tissue, which has been performed in several tissues including the thymus, as well as generating synthetic matrices to support T cell development (188–190). Both of these approaches would require some cellular input to generate a functional thymus; namely the thymic epithelial microenvironment would need to be recapitulated with specific factors or, more likely, cells such as TECs derived from multipotent progenitors or reprogrammed, as above. Moreover, a recent report has demonstrated the efficacy of an artificial pre-thymic niche by implanting a scaffold with the Notch ligand DLL4 that acts as an intermediary between the BM and thymus (191). Although these approaches have shown some promise in preclinical mouse studies (192), further advances need to be made before this can be a viable therapeutic option.

## Conclusion

Enhancing the regenerative capacity of the thymus and increasing thymic output, together with the expansion of the TCR repertoire has immensely beneficial clinical implications. Although there has been extensive progress in the development of multiple therapies targeting thymic regeneration and output, a deeper understanding of key endogenous molecular mechanisms that govern involution and regeneration of the thymus are needed to further the development of clinically translatable therapies.

## Author Contributions

SK and JD wrote, drafted, and edited the manuscript. All authors contributed to the article and approved the submitted version.

## Conflict of Interest

The authors have patents and patent applications around potential therapeutics to promote thymus regeneration, including some listed in this review (IL-22 and BMP4) and others as yet unpublished.
